# Neurological Manifestations of Zika Virus Infection: An Updated Review of the Existing Literature

**DOI:** 10.7759/cureus.80960

**Published:** 2025-03-21

**Authors:** Hasan Saeed, Gohar Rehman, Haseeb Mehmood Qadri, Amna Sohail, Arshaman Ul Haq, Hafiz Zeeshan Sadiq, Shahnila Yasin, Muhammad Asadullah Khalid Rana

**Affiliations:** 1 Pathology, Shifa International Hospital, Islamabad, PAK; 2 Internal Medicine, Allama Iqbal Medical College, Lahore, PAK; 3 General Surgery, Lahore General Hospital, Lahore, PAK; 4 Neurological Surgery, Punjab Institute of Neurosciences, Lahore, PAK; 5 Internal Medicine, Lahore General Hospital, Lahore, PAK; 6 Internal Medicine, Aziz Bhatti Shaheed Teaching Hospital, Gujrat, PAK; 7 Internal Medicine, Nawaz Sharif Medical College, Gujrat, PAK; 8 Internal Medicine, Aziz Fatimah Medical and Dental College, Faisalabad, PAK

**Keywords:** encephalitis, fetal zika virus syndrome, guillain-barrè syndrome, meningitis, microcephaly, neurological manifestations, zika virus

## Abstract

Zika virus (ZIKV) is a neurotropic virus closely linked to other flaviviruses like dengue virus, West Nile virus, yellow fever, and Japanese encephalitis virus. Though initially considered a mild virus, ZIKV gained everybody's attention when the World Health Organization (WHO) declared it a global public health emergency in February 2016. Being considered an important cause of innumerable neurological manifestations and pediatric modality, we aimed to present a comprehensive overview of the neurological details of ZIKV infection. This study reviews the neurological manifestations of ZIKV infection. The Preferred Reporting Items for Systematic Reviews and Meta-Analyses (PRISMA) strategy was employed, along with a combination of keywords, to enlist all articles with data on ZIKV and its neurological manifestations, diagnosis, and treatment. All case reports, case series, and systematic reviews published between 2017 and 2024, focusing on neurological manifestations of ZIKV, were included in this study. Case studies, editorials, letters to the editors, and clinical images were excluded. The search was conducted using Boolean operators "AND" and "OR" on PubMed and Google Scholar. A total of five case reports, one case series, and one systematic review and meta-analysis were included.

Out of 603 patients, the study suggested a male preponderance of 366 patients (62.5%) for ZIKV infection. About 258 patients presented with rash (46.1%), 243 with fever (43.8%), and 134 with dysphagia (36.5%). Neurological signs on examination were limb paresis in 545 (91.1%) patients, areflexia in 401 (88.9%) patients, and tetraparesis in 153 (61%) patients. A significant finding on magnetic resonance imaging (MRI) showed enhancement of the distal cord, conus medullaris, and cauda equina in two cases (0.3%). Serological analysis showed a positive plaque reduction neutralization test (PRNT) in 125 (73.5%) patients. Increased protein levels were identified in 240 (78.7%) cases on cerebrospinal fluid (CSF) analysis. The commonest diagnostic modality utilized was polymerase chain reaction (PCR) in 118 (24.3%) cases. Intravenous immunoglobulins (IVIg) were used for the medical management of 442 patients included in this review (77.4%).

ZIKV is known to cause insidious detrimental effects on the central nervous system regardless of the age of an individual. Being a cause of extreme sensorimotor disability, various preventive and precautionary measures are being undertaken to ensure early diagnosis and prevent prolonged liability on a patient's health. Effective therapeutics including IVIg have paved the way in bringing down the hurdles in the management and cure of the infection.

## Introduction and background

The Zika virus (ZIKV), part of the Flaviviridae family, was first isolated in Uganda in 1947 [1,2]. Initially obscure, it gained global attention during its rapid spread across America from 2013 to 2016, with 440,000 to 1.3 million cases reported in Brazil, leading the World Health Organization (WHO) to declare it a public health emergency. In the 1983s, a distinct strain of the virus spread from Africa to Asia. This Asian strain has led to epidemics in French Polynesia in 2007, 2013, and 2014 [1]. ZIKV is now prevalent in tropical and subtropical regions of 87 countries. The coronavirus disease 2019 (COVID-19) pandemic likely led to underreporting due to overwhelmed healthcare systems [2].

ZIKV is typically transmitted through the bite of an infected *Aedes *mosquito, but it can also spread through sexual contact, blood transfusions, and vertical transmission from mother to fetus. ZIKV infection is often asymptomatic or presents with mild symptoms, such as fever, rash, and joint pain. However, it can lead to severe neurological complications, notably congenital Zika syndrome (CZS), including birth defects like microcephaly, affecting about 80% of cases [3]. The 2013-2014 French Polynesia outbreak provided the first evidence of prenatal transmission; in 2015, ZIKV was found in the amniotic fluid of Brazilian women with microcephalic fetuses, with a 14-fold increase in microcephaly reported among infected mothers [4].

In adults, ZIKV infection has been associated with Guillain-Barré syndrome (GBS), a rare neurological disorder causing symmetric muscle weakness, progressive paralysis, transverse myelitis, and meningoencephalitis [5]. Neuroimaging is crucial for identifying neurological manifestations of ZIKV, including post-contrast enhancement of cranial nerves (particularly facial and trigeminal), intracranial calcifications, and cortical thinning [6].

There is a crucial need to draw attention to the catastrophic neurological manifestations linked with ZIKV infection, which have received remarkably little attention. While ZIKV is commonly associated with moderate symptoms or congenital problems, its capacity to induce serious and possibly fatal neurological diseases in both pediatric patients and adults is highly concerning. Currently, PubMed lacks comprehensive reviews that cover the entire range of neurological manifestations and imaging findings associated with ZIKV. This review sought to address this vacuum by examining these crucial elements in-depth, emphasizing the grave risks posed by ZIKV on the central nervous system and directing future efforts to understand and minimize these risks.

## Review

Methodology

This review incorporates articles published between 2017 and 2024 to put forward a comprehensive overview of the neurological manifestations associated with ZIKV. 

Search Strategy

**Figure 1:**
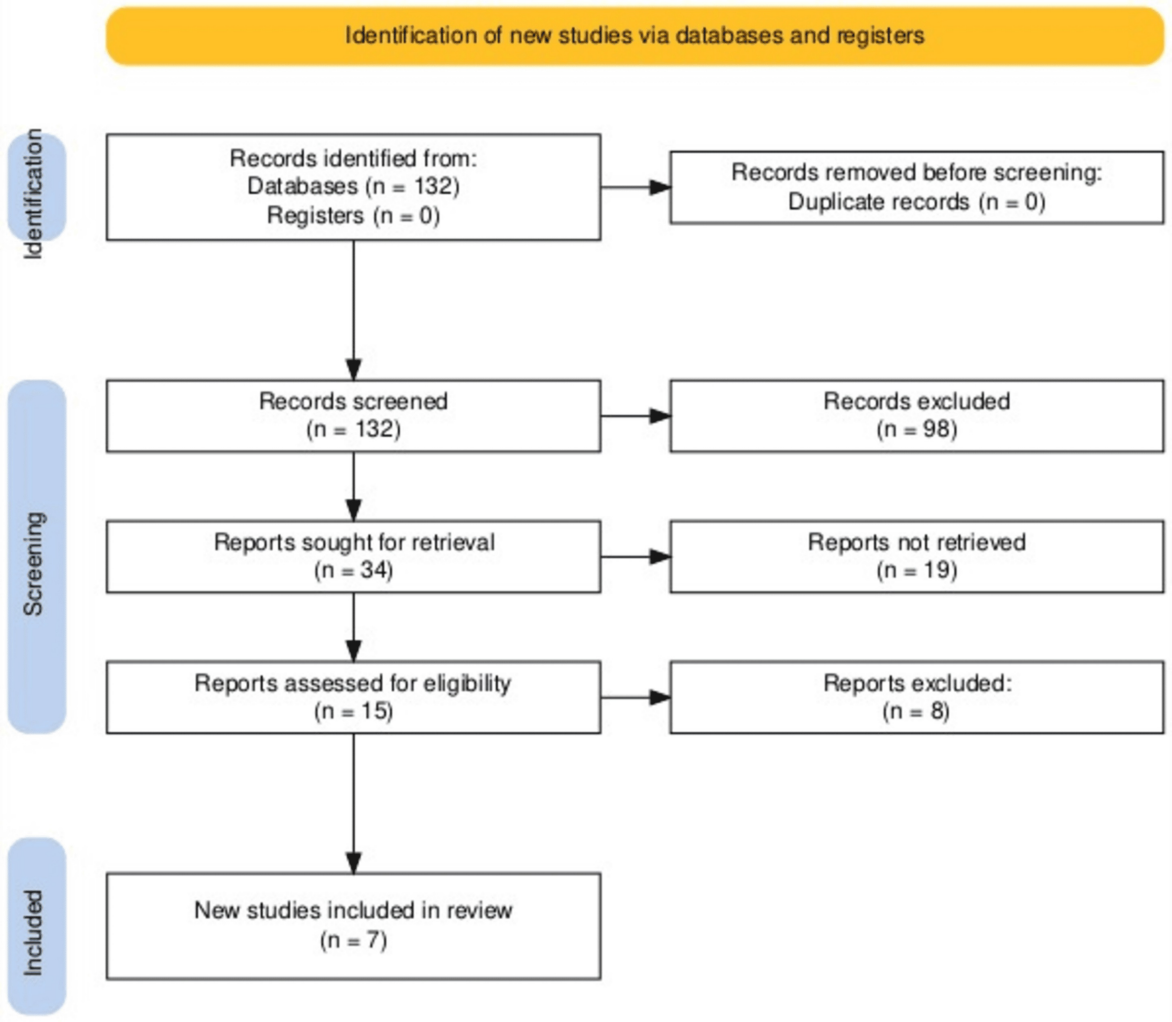
PRISMA flowchart for the search strategy and quality assessment of included studies PRISMA: Preferred Reporting Items for Systematic Reviews and Meta-Analyses

The Preferred Reporting Items for Systematic Reviews and Meta-Analyses (PRISMA) strategy was employed, along with a combination of keywords, to enlist all articles with data on ZIKV (Figure 1). A thorough data collection for this review was done using the database engines PubMed and Google Scholar. Keywords related to ZIKV, its neurological manifestations, diagnoses, and management were utilized to identify the pertinent studies. The search was conducted using Boolean operators ("AND" and "OR") with the following combination of keywords: "Zika virus", "Zika virus infection", "Infection, Zika virus", "Virus infection, Zika", "Zika virus disease”, “Disease, Zika virus", "Virus disease", "Zika", "Zika fever", "Fever, Zika", "ZIKV infection", "Infection, ZIKV", "Congenital Zika syndrome", "Congenital Zika virus infection", "Guillain Barrè syndrome", and "microcephaly".

Inclusion Criteria

Various study designs were included, which ranged from individual case reports and case series to larger reviews and meta-analyses. Only studies published in English and available via free access were considered.

Exclusion Criteria

Conference abstracts, original articles, editorials, letters to the editors, animal and cadaveric studies, and clinical images were excluded.

Data Extraction and Synthesis

The identified articles were selected if they specifically highlighted the neurological impact of ZIKV and were excluded if they were generally based on the microbiology of ZIKV (Figure 1). A thorough search revealed 132 studies. Of these 132 studies, about 32 articles were included by initial screening and 98 were excluded as they did not focus on the neurological impact of ZIKV. The remaining eight were removed because the patient suffered from other infections apart from ZIKV, and their clinical course was based on other infections rather than ZIKV. Finally, seven studies were selected to work on the pre-defined objective. Key information including the study design, sample size, neurological signs, diagnostic modalities, intervention methods, and outcomes was extracted by seven reviewers, who read all the articles independently. The findings were analyzed to identify the common and contrasting themes and scientific gaps in knowledge. Descriptive analysis was utilized to compile the results.

Results

The seven studies included in this review comprised five case reports, one case series, and one systematic review and meta-analysis. The case series included seven cases (1.8%) and the systematic review encompassed 587 cases (97.3%). Almost all of the studies were conducted in Brazil, except for one that was conducted in the United States. Each study incorporated ZIKV-infected cases of variable age and gender, focusing on symptomatology, neurological manifestations, and diagnostic criteria (Tables 1-2).

**Table 1:** The study design and the number of cases involved, with a total of N=603 cases, expressed as a percentage (%)

Study Articles	Number of Studies	Number of Cases, n	Percentage Occurrence, % (n/N)
Case reports	5	5	0.8%
Case series	1	11	1.8%
Systematic review and meta-analysis	1	587	97.3%

**Table 2:** Summaries of the studies included in the review

Study By	Study Title	Year of Publication	Country of Publication
Aspahan et al. [7]	Neuromyelitis optica spectrum disorder associated with Zika virus infection	2019	Brazil
Marinho et al. [8]	Central and peripheral nervous system involvement in Zika virus infection in a child	2019	Brazil
Akrami et al. [9]	The re-emergence of Zika in Brazil in 2020: a case of Guillain-Barré syndrome during the low season for arboviral infections	2020	Brazil
Bentes et al. [10]	Neurologic manifestations of noncongenital Zika virus in children	2021	Brazil
Garcez et al. [11]	Case report: regression of glioblastoma after flavivirus infection	2023	Brazil
Leonhard et al. [12]	Guillain-Barré syndrome related to Zika virus infection: a systematic review and meta-analysis of the clinical and electrophysiological phenotype	2020	Brazil and Netherlands
Pradhan et al. [13]	Case report: Zika virus meningoencephalitis and myelitis and associated magnetic resonance imaging findings	2017	United States

These studies highlighted a clear male preponderance of ZIKV infection, with 366 (62.5%) male cases and 220 (37.5%) female cases (Table 3). The mean age of the patients included was 28.7±15.8 years (mean ± standard deviation) without segregating the pediatric and adult populations.

**Table 3:** Gender distribution of the cases involved in the study, with a total of N=586 cases, expressed as a percentage (%)

Gender	Number of Cases, n	Percentage Occurrence,% (n/N)
Male	366	62.5%
Female	220	37.5%

Even though no well-defined predisposing factors to ZIKV infection were indicated in any of the included studies, asthma, dengue fever, and a history of glioblastoma were noted in 0.2% of the included cases (Table 4).

**Table 4:** Comorbid conditions of the cases involved in the study at the time of presentation, with a total of N=603 cases, expressed as a percentage (%)

Comorbid Conditions	Number of Cases, n	Percentage Occurrence,% (n/N)
Asthma	1	0.2%
Dengue fever	1	0.2%
Glioblastoma	1	0.2%

The total number of cases (N) mentioned in the forthcoming tables varies for each parameter, as one of the studies did not provide a different total number of cases when discussing each parameter.

The rash was most commonly present in 258 (46.1%) cases, followed by fever in 258 (43.8%), dysphagia in 134 (36.5%), arthralgia in 153 (27.6%), and myalgias in 132 (23.3%) patients (Table 5).

**Table 5:** Presenting complaints of the cases involved in the study at the time of presentation, with a total of N=603 cases, expressed as a percentage (%)

Presenting Complaints	Total Number of Cases, N	Number of Cases, n	Percentage Occurrence, % (n/N)
Rash	560	258	46.1%
Fever	555	243	43.8%
Dysphagia	367	134	36.5%
Arthralgia	555	153	27.6%
Myalgia	566	132	23.3%
Dysarthria	297	65	21.9%
Headache	566	112	19.8%
Conjunctivitis	561	99	17.6%
Vomiting	566	66	11.7%
Diarrhea	566	60	10.6%
Nausea	566	59	10.4%
Sensory symptoms	437	33	7.6%
Chest pain	566	28	5.0%
Cough	566	28	5.0%
Diplopia	250	11	4.4%
Ocular pain	560	24	4.3%
Rhinorrhea	566	12	2.1%
Seizures	603	6	1.0%
Gait disability	603	2	0.3%
Paraesthesia	603	2	0.3%
Confusion	603	1	0.2%
Fatigue	603	1	0.2%
Loss of consciousness	603	1	0.2%
Muscle weakness	603	1	0.2%
Neck stiffness	603	1	0.2%
Photophobia	603	1	0.2%
Respiratory distress	603	1	0.2%
Retro-orbital pain	603	1	0.2%
Sore throat	603	1	0.2%
Urinary incontinence	603	1	0.2%
Urinary retention	603	1	0.2%

ZIKV infection can result in significant neurological damage leading to sensorimotor disability. The predominant neurological signs noted were paresis, irrespective of the limb, in 545 (91.1%) patients, aflexia in 401 (88.9%) patients, tetraparesis in 153 (61%) patients, facial palsy in 246 (49%) patients, and sensory deficits in 156 (46.8%) patients (Table 6).

**Table 6:** Neurological signs of the cases involved in the study, with a total of N=603 cases, expressed as a percentage (%)

Neurological Signs	Total Number of Cases, N	Number of Cases, n	Percentage Occurrence, % (n/N)
Any limb paresis	598	545	91.1%
Areflexia	451	401	88.9%
Tetraparesis	267	153	57.3%
Facial palsy	502	246	49.0%
Sensory deficits	333	156	46.8%
Respiratory dysfunction	385	125	32.5%
Bulbar palsy	198	60	30.3%
Paraparesis	267	72	26.9%
Ataxia	333	77	23.1%
Dysautonomia	375	73	19.5%
Ocular palsy	248	22	8.9%
Encephalitis	603	5	0.8%
Meningitis	603	3	0.5%
Ankle clonus	603	2	0.3%
Bilateral Babinski sign	603	2	0.3%
Decreased tendon reflexes	603	2	0.3%
Neuropathic pain	603	2	0.3%
Central pattern nystagmus	603	1	0.2%
Diffuse hyperreflexia	603	1	0.2%
Diminished tactile sensation	603	1	0.2%
Oscillatory vertigo	603	1	0.2%
Spasticity of the lower extremity	603	1	0.2%
Tetraplegia	603	1	0.2%

Non-neurological signs included bulging fontanelle, conjunctival suffusion, high-grade fever, and tachycardia, each displayed in 0.2% of the cases (Table 7).

**Table 7:** Non-neurological signs of the cases involved in the study, with a total of N=603 cases, expressed as a percentage (%)

Non-neurological Signs	Number of Cases, n	Percentage Occurrence, % (n/N)
Bulging fontanelle	1	0.2%
Conjunctival suffusion	1	0.2%
High grade fever	1	0.2%
Tachycardia	1	0.2%

ZIKV can also present as a co-infection with other viruses, particularly in areas where these infections and the vectors responsible for their transmission are prevalent. The chikungunya virus was the most common co-infection, affecting 16 (7.9%) patients, followed by dengue virus in four (1.6%) patients. Dengue and chikungunya co-infection was also identified in six (3.3%) cases (Table 8).

**Table 8:** Co-infection with other viruses in the cases involved in the study, expressed as a percentage (%)

Co-infection With Other Viruses	Total Number of Cases, N	Number of Cases, n	Percentage Occurrence, % (n/N)
Chikungunya virus	203	16	7.9%
Dengue and chikungunya virus co-infection	181	6	3.3%
Dengue virus	251	4	1.6%

A positive plaque reduction neutralization test (PRNT) was the most common serological marker for ZIKV infection, identified in 125 cases (73.5%). This was followed by a raised IgM titer, irrespective of the sample collected for interpretation, in 256 cases (65.5%) (Table 9).

**Table 9:** Immunological assessment of the cases involved in the study, expressed as a percentage (%) PRNT: Plaque reduction neutralization test; CSF: Cerebrospinal fluid

Immunological Assessment	Total Number of Cases, N	Number of Cases, n	Percentage Occurrence, % (n/N)
PRNT	170	125	73.5%
IgM (any sample)	391	256	65.5%
IgM (serum)	390	229	58.7%
IgM (CSF)	127	37	29.1%
IgG	603	1	0.2%

Only one patient had a known case of glioblastoma associated with IDH1 and IDH2 mutations. MRI was used in 0.6% of the cases as a positive diagnostic modality. Enhancement of the distal spinal cord, conus medullaris, and cauda equina was the most significant radiological finding identified in cases exhibiting neurological manifestations, seen in two patients (0.3%), suggesting that radiological investigations serve merely as an aiding tool in assessing the complications of ZIKV infection (Table 10).

**Table 10:** Radiological findings of the cases involved in the study, with a total of N=603 cases, expressed as a percentage (%)

Radiological Findings	Number of Cases, n	Percentage Occurrence, % (n/N)
Enhancement of the distal spinal cord, conus medullaris, and cauda equina	2	0.3%
Cortical edema	1	0.2%
Effacement of sulci	1	0.2%
Hyperintense, non-enhancing lesions at cervical vertebrae C3-C6, thoracic vertebrae T1-T4 and T6-T9, pons, and cerebellar peduncles	1	0.2%
T2 hyperintensity and T1 enhancement of optic nerve	1	0.2%
Periependymal lesion	1	0.2%

Increased protein levels were noted in 240 (78.7%) patients and albuminocytological association was observed in 276 (78.6%) patients, constituting the most common laboratory findings from cerebrospinal fluid (CSF) analysis (Table 11).

**Table 11:** CSF analysis of the cases involved in the study, expressed as a percentage (%) CSF: Cerebrospinal fluid

CSF Analysis	Total Number of Cases, N	Number of Cases, n	Percentage Occurrence, % (n/N)
Increased protein level	305	240	78.7%
Albuminocytological association	351	276	78.6%
Leucocytosis	603	5	0.8%
Oligoclonal bands	603	1	0.2%
Normal	603	1	0.2%

Being a non-specific inflammatory marker, raised serum C-reactive protein (CRP) levels were demonstrated in 11 (1.8%) of the ZIKV-infected cases (Table 12).

**Table 12:** Hematological findings of the cases involved in the study, with a total of N=603 cases, expressed as a percentage (%) CRP: C-reactive protein; ESR: Erythrocyte sedimentation rate

Hematological Findings	Number of Cases, n	Percentage Occurrence, % (n/N)
Raised CRP	11	1.8%
Decreased hemoglobin	1	0.2%
Decreased hematocrit	1	0.2%
Leucocytosis	1	0.2%
Leucopenia	1	0.2%
Raised ESR	1	0.2%

Polymerase chain reaction (PCR) was the principal diagnostic modality used for ZIKV, irrespective of the sample used for testing, in 118 cases (24.3%) (Table 13).

**Table 13:** Diagnostic modality utilized for Zika virus-affected cases involved in the study, expressed as a percentage (%) PCR: Polymerase chain reaction; CSF: Cerebrospinal fluid; ELISA: Enzyme-linked immunosorbent assay

Diagnostic Modality	Total Number of Cases, N	Number of Cases, n	Percentage Occurrence, % (n/N)
PCR (any sample)	486	118	24.3%
PCR (urine)	269	49	18.2%
PCR (serum)	425	43	10.1%
PCR (CSF)	260	24	9.2%
Serology	603	3	0.5%
ELISA	603	1	0.2%
PCR (saliva)	603	1	0.2%
Viral isolation in mice	603	1	0.2%

Intravenous immunoglobulins (IVIg) in 442 (77.4%) patients and plasma exchange in six (1.1%) patients were the predominant medical management strategies utilized for ZIKV-infected cases (Table 14).

**Table 14:** Medical management of the cases involved in the study, expressed as a percentage (%) IVIg: Intravenous immunoglobulins

Medical Management	Total Number of Cases, N	Number of Cases, n	Percentage Occurrence, % (n/N)
IVIg	571	442	77.4%
Both IVIg and plasma exchange	571	12	2.1%
Plasma exchange	571	6	1.1%
Antibiotics	603	4	0.6%
Acyclovir	603	2	0.3%
Steroids	603	2	0.3%
Conservative	603	1	0.2%

One case with a history of glioblastoma underwent surgical treatment (right temporal craniotomy and wedge resection of the tumor followed by microsurgical clipping of the resulting aneurysm) (0.2%).

Walking disability was the most common post-treatment sequela (0.3%), followed by frequent headaches, learning difficulties, delayed development, intense lower back pain, myeloradiculopathy, brain aneurysm, meningoencephalitis, tactile and temperature allodynia, visual impairment, eye pain, and afferent pupillary defect, each encountered in 0.2% of the cases.

After management, about 294 (52.5%) patients remained static, 117 (20.1%) patients' health deteriorated, and 23 (4.6%) died. Only nine (1.5%) patients' health improved (Table 15), highlighting the insidious nature of this viral infection.

**Table 15:** Patient outcomes of the cases involved in the study, expressed as a percentage (%)

Patient Outcomes	Total Number of Cases, N	Number of Cases, n	Percentage Occurrence, % (n/N)
Static	560	294	52.5%
Deteriorated	583	117	20.1%
Died	501	23	4.6%
Improved	603	9	1.5%

Discussion

Epidemiology and Demographics

ZIKV is a mosquito-borne virus notorious for many outbreaks, particularly in Latin American and Caribbean nations since 2007 [1]. In May 2015, the virus spread to Southern and Central America on a pandemic scale [1]. This is comparable to our study in which Brazil reported the greatest number of cases, followed by the Netherlands and the United States of America.

Age and Gender Predilection

Among the seven studies utilized for this systematic review, 62.5% of patients affected by ZIKV were male and 37.5% were female. In contrast, studies conducted on the ZIKV epidemic in 2016 revealed a female predominance, with women accounting for 61% of the total 28,219 reported cases [7]. The aforementioned 2016 study also showed the highest incidence of ZIKV infection among persons aged 20-29 years [7]. This finding is similar to the mean age calculated in our study, which was 28.7±15.8 years.

Presenting Complaints

This study revealed that the most common presenting complaints were rash (46.1%), fever (43.8%), dysphagia (36.5%), arthralgia (27.6%), and myalgia (23.3%), followed by headache (19.8%). This is consistent with another review conducted in Italy, which proposed that ZIKV either presents as a mild infection causing short-term fever, headache, arthromyalgia, maculopapular rash, and sometimes conjunctivitis, or as an asymptomatic disease [3]. Another study also suggested that patients with ZIKV commonly present with acute symptoms like headache, fever, conjunctivitis, myalgia, arthralgia, and rash [14].

Predisposing Factors, Neurological and Non-neurological Manifestations

Following the 2015 ZIKV epidemic in Latin America, there was a considerable increase in patients presenting with microcephaly and GBS, further linking ZIKV to other complications like acute disseminating myeloencephalitis [7]. In this review, the most prevalent neurological symptoms seen post-ZIKV infection were limb paresis (91.1%), areflexia (88.9%), tetraparesis (61%), and facial palsy (49%). A similar picture was observed in a seven-year-old asthmatic child in Brazil who presented with vomiting, headache, and tonic-clonic seizures without any history of epilepsy. Neurological examination revealed clinical myalgia in both lower limbs, with bilaterally reduced reflexes, power, and gait disturbances. He was diagnosed with encephalomyeloradiculitis. Further CSF analysis confirmed ZIKV infection [8]. This highlights that asthma might be a predisposing factor to this infection.

A rare case of neuromyelitis optica was also linked with ZIKV infection, complicated by a preceding dengue virus infection in the patient. This signifies that one viral infection can lead to another viral infection, probably due to variations in the immune status of an individual. Magnetic resonance imaging (MRI) confirmed acute myelitis and optic neuritis with concurrent positive ZIKV PCR [7]. In our study, about 1.6% of patients had co-infection with the dengue virus. Furthermore, a study of the French epidemic in 2013 revealed that out of 42 cases of GBS, 88% reported a history of Flavivirus syndrome [5]. Moreover, dengue virus IgM was positive in 19% of GBS cases, whereas ZIKV IgM was also positive [5]. In our study, respiratory distress due to neurological dysfunction was seen in 32.5% of patients and paraparesis in 27%, similar to a patient diagnosed with GBS and concomitant ZIKV infection. The patient developed ascending paresthesias, followed by paraparesis of lower extremities and urinary incontinence, eventually leading to respiratory distress and dysphagia [9].

ZIKV infection has also been associated with ophthalmological manifestations. One study explained that ZIKV-associated glaucoma can lead to visual field defects and eventually blindness [1]. The most common neuro-ophthalmological sign in our study was ocular palsy (8.9%). Encephalitis and meningitis with concomitant ZIKV infection were seen in 0.8% and 0.5% of the cases, respectively, in our review, similar to a study performed in Colombia that linked ZIKV infection to six cases of pediatric encephalitis [14].

CZS encompasses congenital anomalies acquired by the baby due to maternal ZIKV infection during pregnancy [15]. It includes microcephaly, brain and ocular anomalies, intrauterine growth restriction, congenital contractures, hypertonia with extrapyramidal symptoms, and delayed neurological development [3]. About 90.7% of these cases present with convulsions and severe motor impairment in infancy and early childhood [15]. Approximately 18% of such infants show progressive ventriculomegaly, while 5% have communicating hydrocephalus linked to a higher frequency of seizures and worse neurological disability. A notable percentage of these infants have cerebellar and brainstem hypoplasia as well [6]. In our study, meningitis, encephalitis, and neuropathic pain were the most common pediatric presentations, with one case presenting with bulging fontanelle (0.2%) [10].

Immunological Assessment

This review noted that PRNT was positive in 73.5% of cases, aligning with 90% neutralization in a glioblastoma patient in China [11]. IgM tests were positive in 65.5% of cases, with serum IgM tests showing a 58.7% positivity rate, significantly higher than the 24.3% positive rate for PCR testing. This suggests that IgM tests may be more sensitive than PCR for diagnosing ZIKV, especially in later stages. A 2021 review supports IgM tests’ cost-effectiveness for resource-limited regions [4]. However, Cavalcante et al. found a high false-negative rate for PRNT, with 57% of CZS cases showing negative PRNT results. Another study showed that only 48.5% of quantitative real-time (qRT) PCR-positive mothers were PRNT-positive. Additionally, three out of four ZIKV-related IgM cases had negative PRNT results, questioning PRNT’s reliability. Thus, qRT PCR remains the gold standard test for ZIKV infection, with clinical diagnosis crucial for CZS due to serological test limitations [15]. PCR testing on various sample types emerged as the primary diagnostic tool in this study, with 24.3% of cases testing positive for ZIKV infection. Among the samples, urine showed the highest positivity rate at 18.2%, followed by serum at 10.1%. These results align with another research highlighting PCR's effectiveness on urine and serum for diagnosing ZIKV [1]. PCR testing on CSF samples yielded a 9.2% positivity rate, consistent with Leonhard et al.'s 2020 study, which found only 10 out of 244 cases positive [12]. Although lower than urine and serum, CSF's proximity to the central nervous system offers crucial insights into the virus's impact on the brain and spinal cord.

Radiological Assessment

MRI was used in only 0.6% of patients in this review, indicating that it is not the primary diagnostic tool for ZIKV infection. A 2016 review article notes that, while not definitive, neuroimaging techniques like MRI can provide valuable information regarding ZIKV's effects, particularly in identifying brain anomalies and neurological complications in adults and newborns [16]. Thus, MRI, though not routinely used for diagnosis, is valuable for assessing neurological involvement in ZIKV infection. In this review, the most common radiological finding included enhancement of the distal cord, conus medullaris, and cauda equina, seen in 0.3% of the cases. Less frequent findings, such as cortical edema, sulci effacement, T2 hyperintensity, T1 enhancement of the optic nerve, and periependymal lesions, occurred in 0.2% of cases. This suggests that the impact of ZIKV infection on the spinal cord is relatively rare. Aspahan et al. observed hyperintense, non-enhancing spinal cord lesions on T1-weighted MRI, indicative of acute myelitis [7]. Similarly, Pradhan et al. described extensive T2 fluid-attenuated inversion recovery (FLAIR) hyperintensities in the brain and spinal cord, highlighting the diverse neuroanatomical targets of ZIKV infection [13].

CSF Analysis

CSF analysis showed a high prevalence (78.7%) of increased protein levels in ZIKV-infected patients. This finding aligns with the recognized impact of ZIKV infection on the central nervous system. Similarly, albuminocytological dissociation was observed in 78.6% of cases, further supporting central nervous system involvement. In contrast, case studies on non-congenital ZIKV in children in 2021 did not show pleocytosis in 73% of cases [10]. However, a 2020 study by Leonhard et al. highlighted albuminocytological dissociation in 98% of confirmed Zika cases [12]. Moreover, leucocytosis in CSF was observed in only 0.8% of patients, consistent with another study by Salgado et al., which presented lymphocytic predominant pleocytosis in children with ZIKV-associated encephalitis [17].

Hematological Findings

Our study's hematological findings indicated a low incidence of abnormalities linked to ZIKV infection. Elevated CRP levels were the most frequent, occurring in 1.8% of cases, suggesting a minor inflammatory response. Other abnormalities, such as decreased hemoglobin, hematocrit, leucocytosis, leucopenia, and elevated erythrocyte sedimentation rate, were each found in only 0.2% of cases. This low incidence could be attributed to a minimal systemic cytokine response, as suggested by Salgado et al. (2020) [17]. Marinho et al.'s study also observed hemoglobinemia and elevated CRP on the second hospitalization day, normalizing or nearing normal by day nine [8].

Medical Management

The primary medical management for ZIKV infection was IVIg, utilized in 77.4% of cases. This high usage implies its potential efficacy in alleviating ZIKV effects, likely through immune response modulation, as suggested by Pinto et al., who found that human anti-Zika virus immunoglobulin (ZIKV-Ig) can reduce viral load at higher concentrations via neutralization [18]. In contrast, a case report by Akrami et al. showed no patient improvement despite early IVIg therapy [9]. Combining IVIg with plasma exchange was rare, constituting 2.1% of cases, while plasma exchange alone was used in only 1.1%. Other treatments, including antibiotics, acyclovir, and steroids, were employed even less frequently, ranging from 0.3% to 0.6%.

Patient Outcomes

Most patients (52.5%) experienced static outcomes, showing no significant improvement or deterioration, suggesting that ZIKV infection might result in long-term health consequences for many. Deterioration occurred in 20.1% of cases, indicating the potential for progressive neurological damage. The mortality rate was 4.6%, underscoring the severity of ZIKV infection, particularly with severe neurological involvement. Improvement was observed in only 1.5% of patients, highlighting the challenges in treating long-term neurological complications of ZIKV. The average hospital stay was 21.25 days, and the average follow-up was 364.7 days, indicating the substantial burden on healthcare systems and the necessity for comprehensive care for affected individuals.

Limitations and future directions

The study's reliance on case reports and case series may limit its generalizability. Future research should focus on larger, prospective studies to better elucidate the full spectrum of neurological manifestations associated with ZIKV infection. Additionally, the low reported rates of some findings (e.g., specific radiological features) may be due to underreporting or a lack of systematic investigation rather than true low prevalence. Standardized protocols for investigating ZIKV-associated neurological complications could help address this issue in future studies. To eliminate bias, a quality assessment of included studies was performed using Joanna Briggs Institute Critical Appraisal Checklists for case reports, case series, and systematic reviews (see Tables 16-18 in Appendices) [19,20].

Clinical recommendations

**Figure 2:**
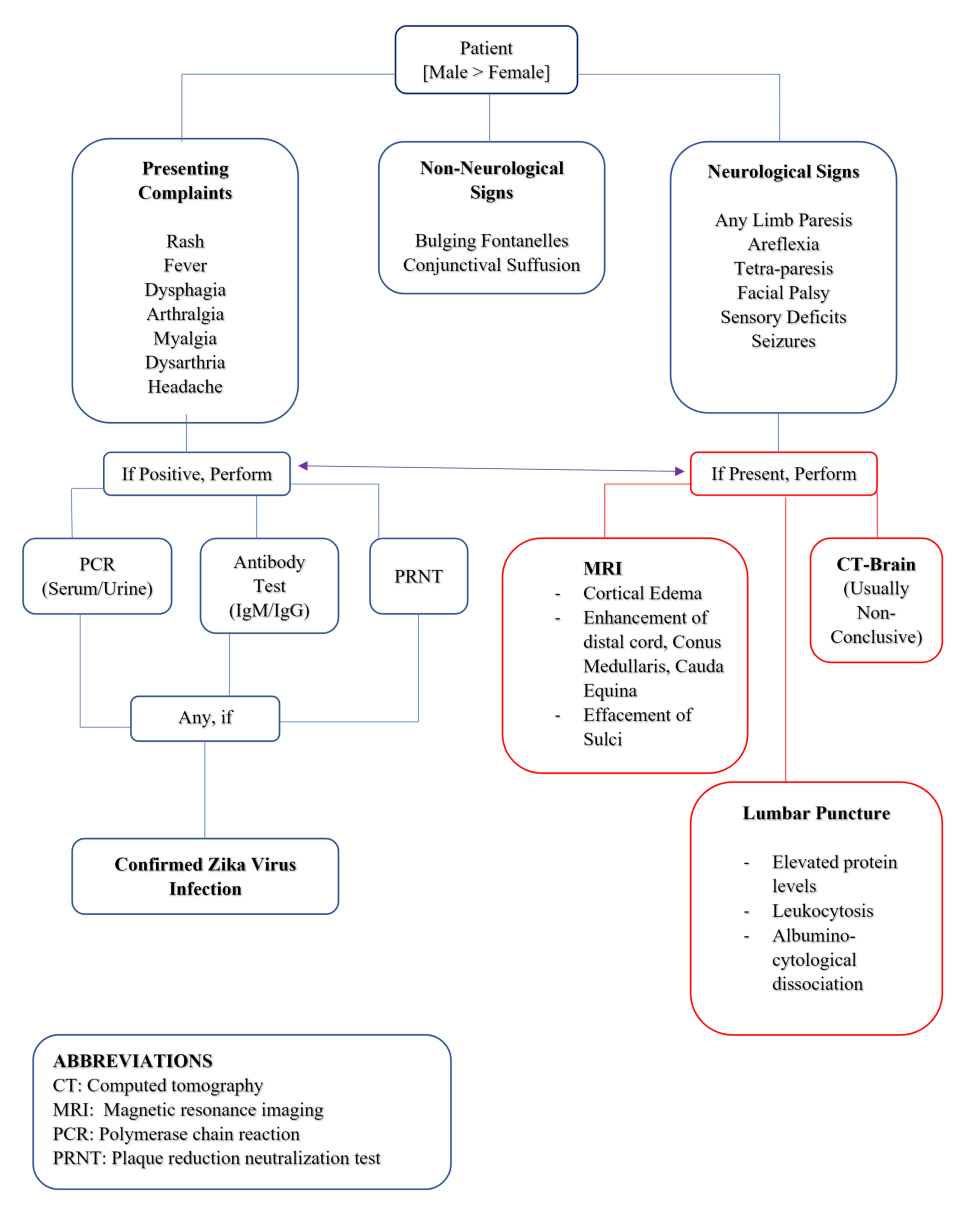
A schematic flowchart for Zika virus infection presenting with neurological manifestations Image Credit: Author Amna Sohail

Based on the study results, healthcare providers should maintain a high index of suspicion for ZIKV-associated neurological complications, particularly GBS, in patients from endemic areas or with relevant travel history. A comprehensive neurological examination is crucial, focusing on limb strength, reflexes, facial nerve function, and sensory deficits. Vigilance for respiratory dysfunction and bulbar palsy is essential, as these may indicate severe cases requiring intensive care. A multimodal diagnostic approach is recommended, combining PRNT with IgM detection in serum, urine, and CSF. CSF analysis should be performed to check for increased protein levels and albuminocytological dissociation. Screening for co-infections, particularly chikungunya and dengue viruses, is advisable. While neuroimaging was not frequently reported, it should be considered in cases with central nervous system symptoms. A multidisciplinary approach involving neurologists, infectious disease specialists, and critical care physicians is optimal for patient management. Long-term follow-up should be implemented to monitor for chronic sequelae, and public health measures should emphasize the prevention of mosquito bites in endemic areas. A schematic flowchart has been added to summarize the clinical manifestations and diagnostic protocol for ZIKV infection presenting with neurological features (Figure 2).

## Conclusions

Post-infectious ZIKV manifestations affecting the neurological system represent an entity with a broad spectrum that is still not fully understood. Much remains to be uncovered regarding viral replication and pathogenesis. This systematic review will play a crucial role in fully comprehending the true extent to which this infection impacts both the central and peripheral nervous systems. It will also aid in understanding the lasting damage it causes, facilitating the development of appropriate diagnostic and therapeutic tools against the virus. Through extensive research, antiviral drugs and safe, effective vaccines can be developed and implemented in time to prevent further complications from this infectious virus and avoid such adverse outcomes. 

## Appendices

**Table 16:** Quality assessment using the Joanna Briggs Institute Critical Appraisal Checklist for the included case reports Y: Yes, N: No, U: Unclear Q1: Were the patient's demographic characteristics clearly described? Q2. Was the patient's history clearly described and presented as a timeline? Q3. Was the current clinical condition of the patient on presentation clearly described? Q4. Were diagnostic tests or assessment methods and the results clearly described? Q5. Was the intervention(s) or treatment procedure(s) clearly described? Q6. Was the post-intervention clinical condition clearly described? Q7. Were adverse events (harms) or unanticipated events identified and described? Q8. Does the case report provide takeaway lessons?

Study By	Q1	Q2	Q3	Q4	Q5	Q6	Q7	Q8	Overall appraisal
Aspahan et al. [9]	Y	Y	Y	Y	Y	Y	Y	Y	Included
Marinho et al. [10]	Y	Y	Y	Y	Y	Y	Y	Y	Included
Akrami et al. [11]	Y	Y	Y	Y	Y	Y	Y	Y	Included
Garcez et al. [14]	Y	Y	Y	Y	Y	Y	Y	Y	Included
Pradhan et al. [17]	Y	Y	Y	Y	Y	Y	Y	Y	Included

**Table 17:** Quality assessment using the Joanna Briggs Institute Critical Appraisal Checklist for the included case series Y: Yes, N: No, U: Unclear Q1. Were there clear criteria for inclusion in the case series? Q2. Was the condition measured in a standard, reliable way for all participants included in the case series? Q3. Were valid methods used for the identification of the condition for all participants included in the case series? Q4. Did the case series have a consecutive inclusion of the participants? Q5. Did the case series have a complete inclusion of the participants? Q6. Was there clear reporting of the demographics of the participants in the study? Q7. Was there clear reporting of the clinical information of the participants? Q8. Were the outcomes or follow-up results of cases clearly reported? Q9. Was there clear reporting of the presenting site(s)/clinic(s) demographic information? Q10. Was statistical analysis appropriate?

Study By	Q1	Q2	Q3	Q4	Q5	Q6	Q7	Q8	Q9	Q10	Overall appraisal
Bentes et al. [13]	Y	Y	Y	Y	Y	Y	Y	Y	Y	Y	Included

**Table 18:** Quality assessment using the Joanna Briggs Institute Critical Appraisal Checklist for the included systematic review Y: Yes, N: No, U: Unclear Q1. Is the review question clearly and explicitly stated? Q2. Were the inclusion criteria appropriate for the review question? Q3. Was the search strategy appropriate? Q4. Were the sources and resources used to search for studies adequate? Q5. Were the criteria for appraising studies appropriate? Q6. Was critical appraisal conducted by two or more reviewers independently? Q7. Were there methods to minimize errors in data extraction? Q8. Were the methods used to combine studies appropriate? Q9. Was the likelihood of publication bias assessed? Q10. Were recommendations for policy and/or practice supported by the reported data? Q11. Were the specific directives for new research appropriate?

Study By	Q1	Q2	Q3	Q4	Q5	Q6	Q7	Q8	Q9	Q10	Q11	Overall appraisal
Leonhard et al. [15]	Y	Y	Y	Y	Y	Y	Y	Y	Y	Y	Y	Included
